# Central Glucocorticoid Administration Promotes Weight Gain and Increased 11β-Hydroxysteroid Dehydrogenase Type 1 Expression in White Adipose Tissue

**DOI:** 10.1371/journal.pone.0034002

**Published:** 2012-03-30

**Authors:** Christelle Veyrat-Durebex, Nicolas Deblon, Aurélie Caillon, Ruth Andrew, Jordi Altirriba, Alex Odermatt, Françoise Rohner-Jeanrenaud

**Affiliations:** 1 Laboratory of Metabolism, Department of Internal Medicine, Faculty of Medicine, University of Geneva, Geneva, Switzerland; 2 Mass Spectrometry Core Laboratory, Queen's Medical Research Institute, Edinburgh, United Kingdom; 3 Division of Molecular and Systems Toxicology, Department of Pharmaceutical Sciences, University of Basel, Basel, Switzerland; Sapienza University of Rome, Italy

## Abstract

Glucocorticoids (GCs) are involved in multiple metabolic processes, including the regulation of insulin sensitivity and adipogenesis. Their action partly depends on their intracellular activation by 11β-hydroxysteroid dehydrogenase type 1 (11β-HSD1). We previously demonstrated that central GC administration promotes hyperphagia, body weight gain, hyperinsulinemia and marked insulin resistance at the level of skeletal muscles. Similar dysfunctions have been reported to occur upon specific overexpression of 11β-HSD1 in adipose tissue. The aim of the present study was therefore to determine whether the effects of central GC infusion may enhance local GC activation in white adipose tissue. Male Wistar and Sprague Dawley (SD) rats were intracerebroventricularly infused with GCs for 2 to 3 days. Body weight, food intake and metabolic parameters were measured, and expression of enzymes regulating 11β-HSD1, as well as that of genes regulated by GCs, were quantified. Central GC administration induced a significant increase in body weight gain and in 11β-HSD1 and resistin expression in adipose tissue. A decrease 11β-HSD1 expression was noticed in the liver of SD rats, as a partial compensatory mechanism. Such effects of GCs are centrally elicited. This model of icv dexamethasone infusion thus appears to be a valuable acute model, that helps delineating the initial metabolic defects occurring in obesity. An impaired downregulation of intracellular GC activation in adipose tissue may be important for the development of insulin resistance.

## Introduction

Glucocorticoids (GCs) influence a wide variety of physiological functions, including food intake, body weight and energy metabolism [1], [2]. Excess of GCs, whether of endogenous origin as in Cushing's syndrome [3], [4] or genetically obese mice and rats [5], [6] is known to promote obesity, insulin resistance, hyperglycemia, and dyslipidemia, characterizing the “metabolic syndrome”. Although a state of hypercorticism is often encountered in idiopathic obesity, circulating cortisol/corticosterone levels can be near normal and even low [7], [8]. These observations do not preclude the presence of increased local GC activity in obesity/insulin resistance [9], [10]. Indeed, intracellular activity of GCs not only depends on their circulating levels, but also on their tissue-specific metabolism by two distinct isoforms of 11β-hydroxysteroid dehydrogenase (11β-HSD), type 1 and type 2 [11], [12], and by expression levels of the GC receptor (GR). 11β-HSD1, expressed in both human and rodent liver [11], adrenal cortex [13] and adipose tissue [14], acts as a low affinity (high *K*_m_ for cortisol/corticosterone) NADP(H)-dependent reductase, leading to increased GC activation [15]. The importance of 11β-HSD1 in adipose tissue is suggested by the observation of a three to four fold increase in 11β-HSD1 reductase activity in adipose tissue of obese patients [16]. Glucose intolerance and muscle insulin resistance are associated with increased adipose tissue 11β-HSD1 activity in obese humans [17], as well as adipose tissue insulin resistance in mice [18]. The physiopathological impact of such an increase has been studied by overexpressing 11β-HSD1 specifically in adipose tissue in mice. Strikingly, these rodents have developed a phenotype similar to the metabolic syndrome, with increased central adiposity, impaired glucose tolerance, hypertriglyceridemia but normal circulating corticosterone levels [19], [20]. Reciprocally, mice lacking 11β-HSD1 are shown to be resistant to the development of the metabolic syndrome and exhibited improved glucose tolerance, despite high basal plasma corticosterone levels [10].

On the other hand, 11β-HSD2, mainly expressed in aldosterone-target tissues such as the distal nephron and colon, is a high affinity 11β-dehydrogenase that catalyzes rapid inactivation of GCs. Interestingly, 11β-HSD2 was recently reported to be expressed in adipocytes and the stroma-vascular fraction of adipose tissue of obese rats and humans [21], [22], suggesting that this enzyme could play an important role in intracellular GC inactivation and availability for GR in this tissue [23].

Altogether, these observations strongly suggest that adipose tissue-specific modulation of GC production is a key determinant of metabolic homeostasis, particularly during periods of enhanced weight gain and fat deposition. Interestingly, we have previously showed that infusion of the synthetic GC dexamethasone results in increased food intake and a rapid body weight gain, hyperinsulinemia, hypertriglyceridemia, hyperleptinemia and skeletal muscle insulin resistance upon central, but not peripheral administration in Sprague Dawley (SD) rats [24], [25]. Such deleterious effects of elevated central GCs are suggested to be mediated by a concomitant increase in hypothalamic neuropeptide Y (NPY) and decrease in corticotropin-releasing hormone (CRH) content, in line with the presence of hypocorticosteronemia due to the feedback inhibition of the hypothalamo-pituitary-adrenal (HPA) axis [24], [25]. NPY is indeed a potent orexigenic factor that also affects peripheral metabolism by increasing lipogenic pathways and promoting muscle insulin resistance. The aim of the present study was therefore to investigate whether the deleterious metabolic effects of central dexamethasone infusion also involve modulation of adipose tissue 11β-HSD type 1 and 2 expression. This was studied in two different strains of rats, displaying different susceptibilities to the development of obesity. The gene expression levels of resistin, an adipocyte-derived factor that impairs insulin action on glucose metabolism [26], [27], [28], as well as promotes dyslipidemia in rodents [29] was also determined. Indeed, it has been shown that GCs upregulated resistin expression, both *in vivo* and *in vitro*, although this effect might not be exerted directly, *via* a glucocorticoid-responsive element [30]. Finally, the central mediation of adipose tissue 11β-HSD1 expression changes was assessed by determining the effects of peripherally administered dexamethasone on metabolic parameters. We showed that central GC administration induced a significant increase in body weight gain and 11β-HSD1 and resistin expression in adipose tissue, while these changes were not observed when dexamethasone was delivered peripherally. Such centrally elicited effects may thus be important for the development of insulin resistance.

## Methods

### Ethics Statement

All procedures were performed in accordance with and approved by the Institutional Ethical Committee of Animal Care in Geneva and Cantonal Veterinary Office (experiment ID 1034/3025/2).

### Animals

Male Wistar (200–225 g) and SD (200–225 g) rats were purchased from Charles River (L'Arbresle, France) and housed under controlled temperature (23°C) and lighting (light on: 0700–1900 h). They were allowed free access to water and a standard laboratory diet (RMI, Hersteller, Essex, UK) (14.7% proteins, 2.6% fat, 68.0% carbohydrates). Body weight and food intake were recorded in the morning (0830–0930 h).

### Intracerebroventricular (icv) infusion of dexamethasone

After one week of acclimatization, rats were individually housed and submitted to surgical procedures. They were anesthetized with intramuscular injection of ketamine-xylasine (Ketalar®-Rompun®, Parke-Davis and Bayer, Leverkusen, Switzerland) at 40 and 9 mg/kg, respectively, and implanted with a cannula in the right lateral cerebral ventricle [31]. After one week of recovery, the drinking response to icv injection of Angiotensin II (5 ng/µl) (Novabiochem, Läufelfingen, Switzerland) was measured to confirm the correct placement of the cannula. Osmotic minipumps (Alzet®, model 2001, Alza Corporation, Cupertino, CA) delivering either the vehicle (0.9% NaCl) or dexamethasone diluted in 0.9% NaCl (Aacidexam®, N.V. Organon, Oss, Netherland) were then subcutaneously implanted and connected to the icv cannula *via* a polyethylene catheter, as previously described [31]. From results obtained on the body weight (BW) gain, after a pilot dose-response study (2.5 and 5 µg/day), a dose of 5 µg/day was finally chosen, as previously published [25]. Wistar rats were treated for 3 days and SD rats for 2 days to obtain similar body weight gains. Animals were then sacrificed in the fed state, using isoflurane anesthesia (Halocarbon Laboratories, River Edge, NJ) and rapid decapitation between 0900 and 1300 h. Blood samples were collected into EDTA-coated tubes, centrifuged and plasma stored at −20°C until use. Tissues were rapidly removed, freeze-clamped, and stored at −80°C.

### Peripheral (sc) infusion of dexamethasone

After one week of adaptation, SD rats were individually housed and submitted to surgical procedures. They were briefly anesthetized with isoflurane and osmotic minipumps (Alzet®, model 1003D) delivering either the vehicle (0.9% NaCl) (control group, *n* = 8) or 5 µg/day dexamethasone (*n* = 9) were subcutaneously implanted for two days. Rats were sacrificed by isoflurane anesthesia and rapid decapitation between 0900 and 1200 h. Blood samples and tissues were collected as described above.

### Indirect calorimetry (LabMaster)

Different metabolic parameters, spontaneous activity, as well as food and drinking behaviors were measured using a 12-cage LabMaster indirect calorimetry system (TSE Systems GmbH, Berlin, Germany) of the Small Animal Phenotyping Core facility (CMU, University of Geneva, Geneva), under controlled temperature (22±1°C) and lighting (12 h light-dark cycle). The calorimetry system is an open-circuit determining O_2_ consumption (ml/h/kg), CO_2_ production (ml/h/kg), respiratory exchange rate (RER = *V*CO_2_/*V*O_2_, where *V* is volume), and heat produced by the animal (kcal/h/kg). Detection of animal location and movements is performed with infrared sensor pairs arranged in strips for horizontal activity, discriminating between ambulatory and fine movements. The LabMaster also consists of a combination of highly sensitive feeding and drinking sensors for automated online measurements. Before recording, animals were allowed a 14-day acclimatization period in training cages. Measurements were performed over the 48 h of saline or dexamethasone infusion.

### Body composition

An EchoMRI-700 quantitative nuclear magnetic resonance analyzer (Echo Medical Systems, Houston, TX) was used to measure total fat and lean body mass at the end of the treatments. In addition, various white adipose tissue depots (inguinal, epididymal, perirenal and mesenteric) were carefully dissected and weighted after sacrifice.

### Plasma measurements

Plasma glucose was measured by the glucose oxidase method (Roche Diagnostics GmbH, Rotkreuz, Switzerland). Plasma nonesterified fatty acids (NEFA) and triglycerides (TG) were determined using Wako Chemicals GmbH (Neuss, Germany) and bioMérieux (Marcy l'Etoile, France) commercial kits, respectively. Plasma corticosterone (Immunodiagnostic systems Ltd, Boldon, UK) and leptin (Linco Research Inc., St Charles, MO) levels were determined using a double antibody RIA with a 3% intra-assay coefficient of variation, and plasma insulin levels were measured by RIA, as previously described [32]. In one of the experiments, plasma corticosterone and 11-dehydrocorticosterone were also measured by LC-MSMS (Department of Pharmaceutical Sciences, University of Basel). Plasma dexamethasone levels after icv treatment in Wistar rats were measured by mass spectrometry (Centre for Cardiovascular Science, The Queen's Medical Research Institute, Edinburgh, UK). Briefly, dexamethasone was quantified by liquid chromatography tandem mass spectrometry using as Surveyor HPLC, equipped with a Allure biphenyl column (5 µm, 100×4.6 mm; Thames Restek, Buckinghamshire, UK) linked to a TSQ Quantum mass spectrometer (Thermo Electron, Hemel Hempstead, UK) operated in electrospray mode (source temperature 400°C, spray voltage 3.5 kV, argon pressure 1.5 Pa) as reported before [33]. Steroids were eluted using a mobile phase of 60∶40 (methanol: ammonium acetate (5 mM), 0.5 ml/min, 30°C) and quantified *versus* a calibration curve. Mass transitions monitored were dexamethasone (*m*/*z* 393→147), D4-dexamethasone (397→377 and →359).

### Total adipose tissue lipids and hepatic TG content

For the determination of total lipids in inguinal adipose tissue, weighed quantities of frozen tissue (≈200 mg) were powdered under liquid N_2_ and extracted overnight at 4°C in 5 ml chloroform/methanol (2∶1 vol/vol). After filtration, extracts were added with 1 ml sterile water and centrifuged (3,000 *g*, 10 min). They were washed twice by adding 1.5 ml of upper phase washing buffer (3.1% (vol/vol) chloroform, 49% methanol, CaCl_2_-2H_2_O 1.77 mM), followed by centrifugation (3,000 *g*, 10 min) and discarding of the aqueous phase. A defined volume was then transferred into pre-weighted glass tubes and evaporated to dryness with N_2_. Lipid content was determined by weighing the tubes. For the determination of TG content [34], weighed quantities of frozen tissue (≈100 mg) were powdered under liquid N_2_ and extracted overnight at 4°C in 6 ml chloroform/methanol (2∶1 vol/vol). Phase separation was obtained after addition of 1 ml H_2_SO_4_ (1 M) and centrifugation (200 *g*, 10 min). The upper phase was discarded and the lower phase washed by a further addition of 1 ml H_2_SO_4_. Phospholipids were then removed by adding silicic acid previously activated by heating (150°C, 2 h) and centrifugation (200 *g*, 10 min). Two ml of the lower organic phase were then removed and evaporated under N_2_. The resulting pellet was suspended in 200 µl assay buffer (Tris 0.4 M, NaCl 0.2 M, EDTA 10 mM, bovine serum albumin 10 mg/ml, 1% Triton X-100, pH 8.4), and triglyceride content was determined by colorimetric enzymatic analysis, as above.

### Tissue processing and real-time RT-PCR

Total RNAs were extracted from frozen tissue using a single-step extraction with Trizol reagent (Sigma-Aldrich, Buchs, Switzerland). RNA integrity was assessed by electrophoresis on a 1% agarose gel, and concentration was determined by spectrophotometry. A quantity of 2.5 µg of total RNAs was used for RT with random hexamers (Microsynth, Geneva, Switzerland), dNTPs (Promega Corporation, Madisson, WI, USA), RNasin as an RNase inhibitor (Promega Corporation), and the M-MLV-RT enzyme kit (Invitrogen, Basel, Switzerland). Amplification was either performed from 64 ng cDNA (2 µl of RT), using the SYBR green I DNA Master kit (Roche Diagnostics, Mannheim, Germany), according to the LightCycler™ standard protocol (Roche Molecular Biochemicals, Basel, Switzerland), or from 12.5 ng cDNA (0.4 µl of RT) using the SYBR® green PCR Master Mix (Applied Biosystems, Warrington, UK) on an ABI7500 machine (Applied Biosystems, Foster City, CA). According to the system used to measure mRNA expression, primers were either designed with the Primer3 and PrimerExpress software (Applied Biosystems) (Table S1) and synthesized by Microsynth (Balgach, Switzerland), Eurogentec (Seraing, Belgium), or provided by Applied Biosystems. Primers were used at a final concentration of 200 to 500 nM. Results were normalized to expression levels of tissue-specific housekeeping genes: ribosomal protein S29 (RPS29) (white adipose tissue), β-actin (liver, white adipose tissue), glyceraldehyde-3-phosphate dehydrogenase (GAPDH), cyclophilin A (liver, white and brown adipose tissue) and 36B4 (hypothalamus).

### Western blot

Frozen tissues were mechanically homogenized in ice-cold RIPA buffer (100 mM Tris, 2% NP40, 0.2% SDS, 0.3 M NaCl and 1% sodium deoxycholate, pH 7.5) containing protease inhibitors (Complete Mini, Roche Diagnostics). Proteins were separated by SDS-10% polyacrylamide gel electrophoresis. After a blocking period (milk 5% TBS tween 0.1%, 1 h), blots obtained after transfer on nitrocellulose membranes were incubated with purified home-made anti-PEPCK [35], anti-11β-HSD1 (Chemicon International Inc., Billerica, MA), anti-C/EBPα, anti-C/EBPβ (Santa Cruz Biotechnology, Inc., Santa Cruz, CA), anti-C/EBPβ phosphorylated on ser^105^ (P-C/EBPβ) (Cell Signaling Technology, Inc., Beverly, MA) and anti-actin (Chemicon International Inc.) antibodies overnight at 4°C. Antibodies against 11β-HSD1 and PEPCK were kindly provided by Dr. Karen E. Chapman (Queen's Medical Research Institute, Edinburgh, UK) and Dr. Ildiko Szanto (Centre Médical Universitaire, Geneva, Switzerland), respectively. Detection was performed using horseradish peroxidase-conjugated secondary antibodies (BIO-RAD Laboratories, Hercules, CA) and an enhanced chemiluminescence (ECL) detection system (Amersham Biosciences). The results were then quantified using the ChemiDoc™ XRS from BIO-RAD and the Quantity One™ software.

### Statistical analyses

Results are expressed as means ± SEM. The Levene test was used to check for the equality of variance among groups (SPSS Inc., Chicago, IL). To assess the effects of treatment, groups' comparison was performed using parametric (Student's *t* test), and non-parametric (Mann-Whitney test) tests when normality and equal variance tests failed. Effect of time was analyzed using one-way repeated measures ANOVA (Student-Newman-Keuls). Linear regression models were used to predict correlations between expression levels of enzymes, using values of the control and the treated groups. Statistical significance was established at *P*<0.05.

## Results

### Stimulation of 11β-HSD1 expression and lipogenesis in Wistar rats after icv dexamethasone infusion

**Figure 1:**
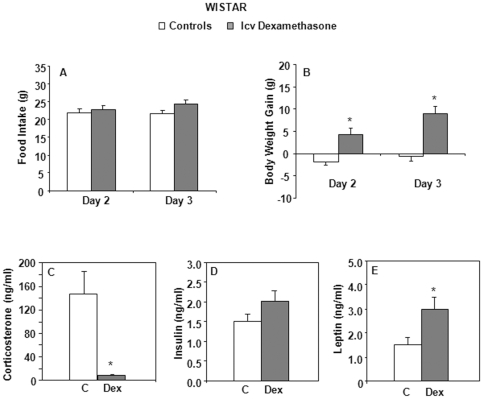
Food intake, body weight gain, plasma corticosterone, insulin and leptin levels of Wistar rats. Central (icv) dexamethasone infusion for 3 days. A) Results are expressed as food ingested (in grams) during the last two days of icv infusion. B) Results represent body weight gain (in grams) measured after 2 and 3 days of icv infusion. C to E) Plasma levels of corticosterone, insulin and leptin. Mean ± SEM of *n* = 7 rats per group. *P<0.05 compared to vehicle-infused control rats (Student's *t* test and Mann-Whitney test).

We first tested the effect of intracerebroventricular (icv) infusion of dexamethasone in Wistar rats, a strain known for its propensity and homogeneity to develop age-dependent obesity [36]. No significant modification of daily food intake was observed in treated animals (Fig. 1A). Only cumulative food intake over the 3 days of the experiment was increased (62.6±2.0 vs. 69.3±1.5 g in control and dexamethasone-treated rats, respectively, *P* = 0.019). Body weight gain was also significantly enhanced (day 2: *P* = 0.007 and day 3: *P*<0.001) (Fig. 1B). As expected, corticosteronemia was markedly reduced in dexamethasone-treated animals (*P* = 0.001) (Fig. 1C), while leptinemia increased (*P* = 0.035) (Fig. 1E). As shown in Fig. 1D and Table 1, plasma insulin, glucose, NEFA and TG concentrations were unaltered by central dexamethasone infusion. Measurements of plasma dexamethasone levels at the end of the experiment revealed a low concentration of the drug (2.94±0.68 ng/ml), far below normal plasma corticosterone levels (Fig. 1C), indicating the absence of leakage from the cerebrospinal fluid into the peripheral circulation.

**Table 1:** Effects of dexamethasone treatment on metabolic parameters.

	Controls	Dex 5 µg/day
**Icv treated Wistar rats**		
Pre-treatment food intake (g)	22.1±2.3	22.9±1.1
Post-treatment body weight (g)	344.6±1.1	336.5±5.9
Glucose (mg/L)	143.6±5.5	146.6±10.4
NEFA (mmol/L)	0.22±0.08	0.65±0.21
TG (mg/dL)	80.3±15.8	56.2±7.7
**Icv treated SD rats**		
Pre-treatment food intake (g)	25.4±0.6	20.2±0.9
Post-treatment body weight (g)	320.5±7.0	338.9±7.5
Glucose (mg/dL)	131.3±2.3	127.7±4.7
NEFA (mmol/L)	0.44±0.06	0.32±0.02
TG (mg/dL)	98.5±14.3	87.7±6.7
Lipid WAT (mg/g tissue)	270.9±37.3	302.0±36.5
Liver TG (mg/g tissue)	8.7±1.2	8.7±0.4
**Sc treated SD rats**		
Glucose (mg/dL)	187.1±15.9	203.4±6.8
NEFA (mmol/L)	0.12±0.06	0.31±0.05*
TG (mg/dL)	86.6±30.6	119.3±39.8

Values represent mean ± SEM of *n* = 7–10 rats per group.

* *P*<0.05 in comparison to control rats, using Student's *t* test.

**Figure 2:**
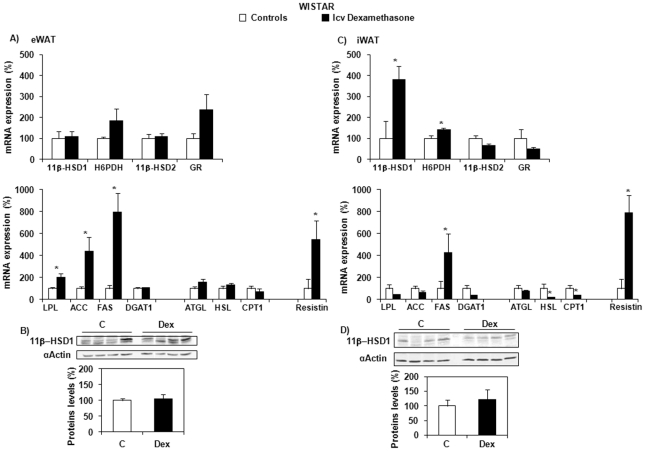
mRNA and protein expression of different markers of GC and lipid metabolism in Wistar rats. Effect of central (icv) dexamethasone infusion for 3 days in epididymal (eWAT) and inguinal (iWAT) white adipose tissue. A and C) Results are expressed as percent of relative mRNA expression compared to that obtained in control rats (100%). The analysis was performed in duplicate and the results were normalized with RPS29 expression. Mean ± SEM of *n* = 7 rats per group. *P<0.05 compared to vehicle-infused control rats (Student's *t* test). B and D) Representative Western blots of 11β-HSD1 (n = 4–5 per group). Quantification was performed using the ChemiDoc™ XRS and the Quantity One™ software.

In epididymal white adipose tissue (eWAT), chosen to represent an intraperitoneal fat pad, neither the mRNA (Fig. 2A), nor the protein (Fig. 2B) expression of 11β-HSD1 was modified by dexamethasone treatment. However, the expression of lipogenic enzymes, such as lipoprotein lipase (LPL), acetylCoA carboxylase (ACC) and fatty acid synthase (FAS), as well as that of resistin were significantly increased. In the inguinal WAT (iWAT), chosen to represent a subcutaneous fat pad, a marked increase in 11β-HSD1 mRNA (4-fold) (*P* = 0.034) (Fig. 2C), with no change in protein expression (Fig. 2D) was observed in the dexamethasone-treated group. This was accompanied by elevated expression of H6PDH (*P* = 0.037), FAS (*P* = 0.008) and resistin (*P* = 0.045) (Fig. 2C). Of note, mRNA expression of enzymes implicated in lipolysis and lipid utilization, such as hormone sensitive lipase (HSL) and carnitine palmitoyltransferase type 1 (CPT1), was significantly decreased (*P* = 0.017 and *P* = 0.004, respectively). In both the intraperitoneal and the subcutaneous fat depots, the expression of 11β-HSD2 and GR was unaffected by central dexamethasone infusion.

Regarding the discrepancy between dexamethasone-induced 11β-HSD1 expression in intraperitoneal and subcutaneous adipose tissue, it is interesting to note that the gene was substantially more expressed in the former than in the latter fat depot (*Ct* of 23.86±0.52 and 32.34±2.31, respectively; *P* = 0.011).

In the liver, the mRNA expression of 11β-HSD1 and H6PDH, as well as the mRNA and protein expression of PEPCK, the key enzyme for gluconeogenesis, were unaltered by the treatment (data not shown).

### Stimulation of 11β-HSD1 expression and lipogenesis in SD rats after icv dexamethasone infusion

**Figure 3:**
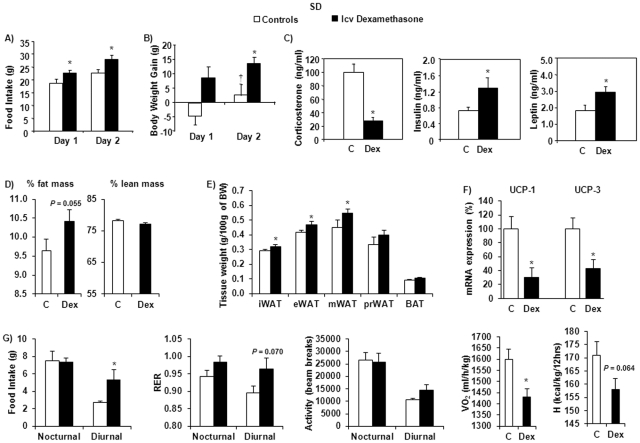
Food intake, body weight gain, plasma corticosterone, insulin and leptin levels, as well as metabolic parameters and locomotor activity of SD rats. Effects of central (icv) dexamethasone infusion for 2 days. A) Results are expressed as food ingested (in grams) each day of icv infusion. B) Results represent body weight gain (in grams) measured after 1 and 2 days of icv infusion. C) Plasma levels of corticosterone, insulin and leptin. D) Body composition (% of fat and lean mass) obtained using EchoMRI700™. E) Weight (g/100 g of BW) of inguinal (iWAT), epididymal (eWAT), mesenteric (mWAT) and perirenal (prWAT) white adipose tissue and brown adipose tissue (BAT). F) Results are expressed as percent of relative mRNA expression compared to that obtained in control rats (100%) and normalized with cyclophilin A expression. Mean ± SEM of *n* = 6–8 rats per group. G) Food intake, respiratory quotient (RER), total activity, VO_2_ and heat (H) production measured using indirect calorimetry (LabMaster). Values are mean ± SE of 5 animals per group. ^†^ *P*<0.05 compared to day 1, using one-way repeated measures ANOVA (Student-Newman-Keuls test). *P<0.05 compared to vehicle-infused control rats (Student's *t* test).

The effects of icv dexamethasone infusion were then studied in SD rats, a strain described as having different susceptibility to obesity development [37], [38], although with some heterogeneity. The treatment induced a significant increase in food intake and body weight gain compared to controls (Figs. 3A and 3B). The expected decrease in corticosteronemia (*P*<0.001) was accompanied by elevated basal plasma insulin (*P* = 0.047) and leptin (*P* = 0.046) levels (Fig. 3C). These results are in line with and extend previously published data showing that icv infusion of GCs results in the induction of increased body weight gain and insulin resistance in SD rats [25]. Plasma glucose, NEFA and TG concentrations, as well as lipid content in inguinal WAT and TG content in the liver were, however, not significantly modified by this short period of dexamethasone treatment (Table 1).

To evaluate the qualitative nature of the dexamethasone-induced increase in body weight, another experiment was performed to measure body composition using an EchoMRI analyser. A tendency toward an increase in percent fat mass was observed in dexamethasone-treated animals (Fig. 3D). At the end of these experiments and following sacrifice, iWAT, eWAT, perirenal (prWAT) and mesenteric (mWAT) adipose tissue depots were dissected, and weighted. Both the subcutaneous iWAT and the intraperitoneal (eWAT, mWAT) fat pads were significantly increased by the dexamethasone treatment (Fig. 3E).

Various metabolic parameters and locomotor activity were also determined. Online food intake measurements allowed to observe that the increased food intake of dexamethasone-treated rats was due to increased food consumption during the diurnal period (Fig. 3G). No modification of the RER was observed during the night. However, the diurnal RER tended to be higher in dexamethasone-treated than in control animals, suggesting decreased lipid mobilization as an energy source. No differences in physical activity, neither ambulatory nor fine, were noticed between the two groups. Recording of VO_2_ and heat (H) production revealed lower values in dexamethasone-treated rats compared to controls, reflecting lower energy expenditure and a trend towards decreased thermogenesis (Fig. 3G). Such a trend was consistent with the observation of decreased mRNA expression of the two uncoupling proteins, UCP1 (*P* = 0.015) and UCP3 (*P* = 0.017) in brown adipose tissue (Fig. 3F), as previously described [11].

Confirming the already described involvement of NPY neurons in the mediation of the central dexamethasone effects [24], mRNA expression levels of NPY were 2.4 fold-increased in hypothalami of treated animals (100.0±65.3 and 241 .1±73.2% for control and treated animals, respectively, *P* = 0.044).

**Figure 4:**
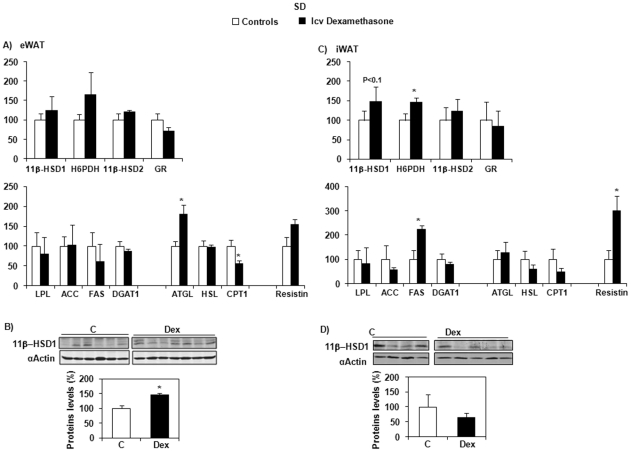
mRNA and protein expression of different markers of GC and lipid metabolism in SD rats. Effects of central (icv) dexamethasone infusion for 2 days in epididymal (eWAT) and inguinal (iWAT) adipose tissue. A and C) Results are expressed as percent of relative mRNA expression compared to that obtained in control rats (100%). The analysis was performed in duplicate and the results are normalized with RPS29 expression. Mean ± SEM of *n* = 7–8 rats per group. *P<0.05 compared to vehicle-infused control rats (Student's *t* test). B and D) Representative Western blots of 11β-HSD1 (n = 6–7 per group). Quantification was performed using the ChemiDoc™ XRS and the Quantity One™ software. *P<0.05 compared to vehicle-infused control rats (Student's *t* test).

In eWAT, mRNA expression of 11β-HSD1 was not modified by dexamethasone treatment (Fig. 4A), but protein expression was significantly increased (*P* = 0.003) (Fig. 4B). In iWAT, 11β-HSD1 mRNA expression tended to increase (Fig. 4C), with no change in protein levels (Fig. 4D). H6PDH, FAS and resistin expression was also significantly enhanced by dexamethasone (*P* = 0.026, *P* = 0.048, and *P* = 0.014, respectively), H6PDH being strongly correlated with 11β-HSD1 expression (r^2^ = 0.507, *P* = 0.004) (Fig. 4C). As observed in Wistar rats, 11β-HSD1 was substantially more expressed in eWAT than in iWAT (*Ct* of 21.99±0.30 and 24.62±0.39, respectively; *P* = 0.0001). In both these depots, neither the mRNA expression of 11β-HSD2, nor that of GR was modified by dexamethasone infusion.

### Partial compensatory mechanism in the liver of SD rats after icv dexamethasone infusion

**Figure 5:**
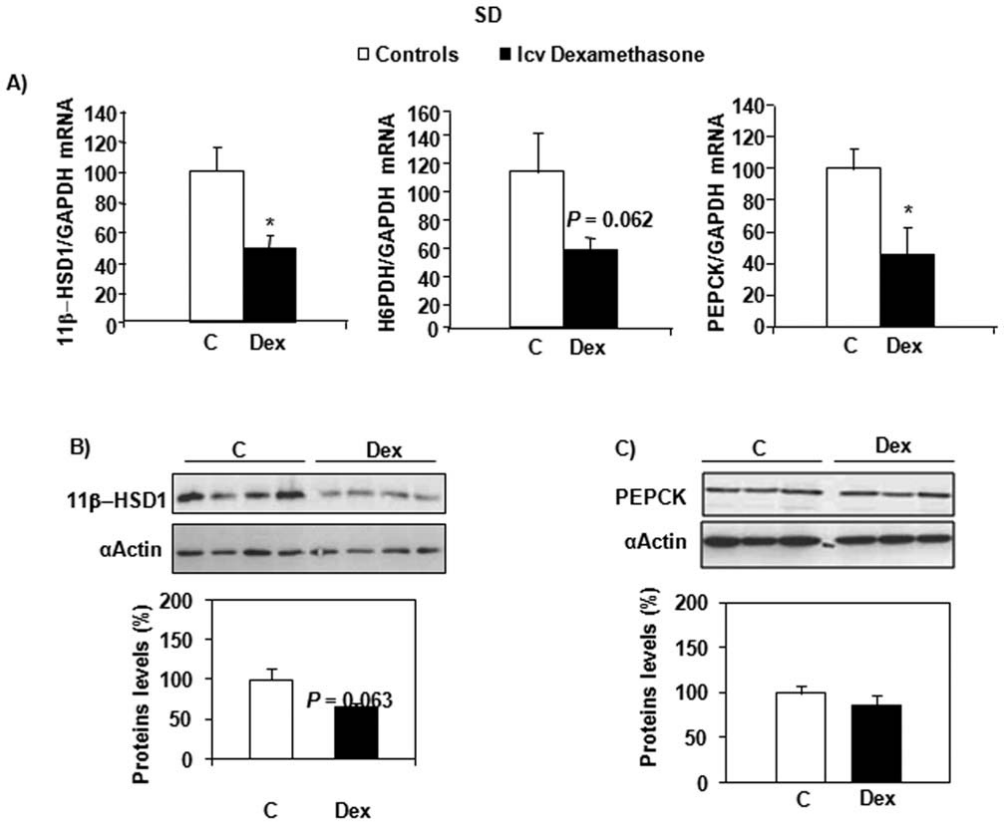
mRNA and protein expression of markers of GC action and gluconeogenesis in the liver of SD rats. Effects of central (icv) dexamethasone infusion for 2 days. A) Results are expressed as percent of relative mRNA expression compared to that obtained in control rats (100%). The analysis was performed in duplicate and the results were normalized with GAPDH expression. Mean ± SEM of *n* = 7–8 rats per group. *P<0.05 compared to vehicle-infused control rats (Student's *t* test). B and C) Representative Western blots of 11β-HSD1 and PEPCK (n = 4–5 per group). Quantification was performed using the ChemiDoc™ XRS and the Quantity One™ software.

In contrast to what was observed in Wistar rats, a significant decrease in 11β-HSD1 mRNA expression was observed in the liver of dexamethasone-treated SD rats (*P* = 0.007), with a similar expression for H6PDH (Fig. 5A), both parameters being highly correlated (r^2^ = 0.692, *P* = 0.001). 11β-HSD1 protein expression also tended to decrease (Fig. 5B). In addition, PEPCK mRNA expression was reduced in dexamethasone-treated rats (*P* = 0.016) (Fig. 5A) and there was a significant correlation between PEPCK and 11β-HSD1 expression in this tissue (r^2^ = 0.353, *P* = 0.032). PEPCK protein levels were, however, unaltered by central dexamethasone infusion (Fig. 5C).

### Implication of C/EBPα and C/EBPβ after icv dexamethasone infusion

**Figure 6:**
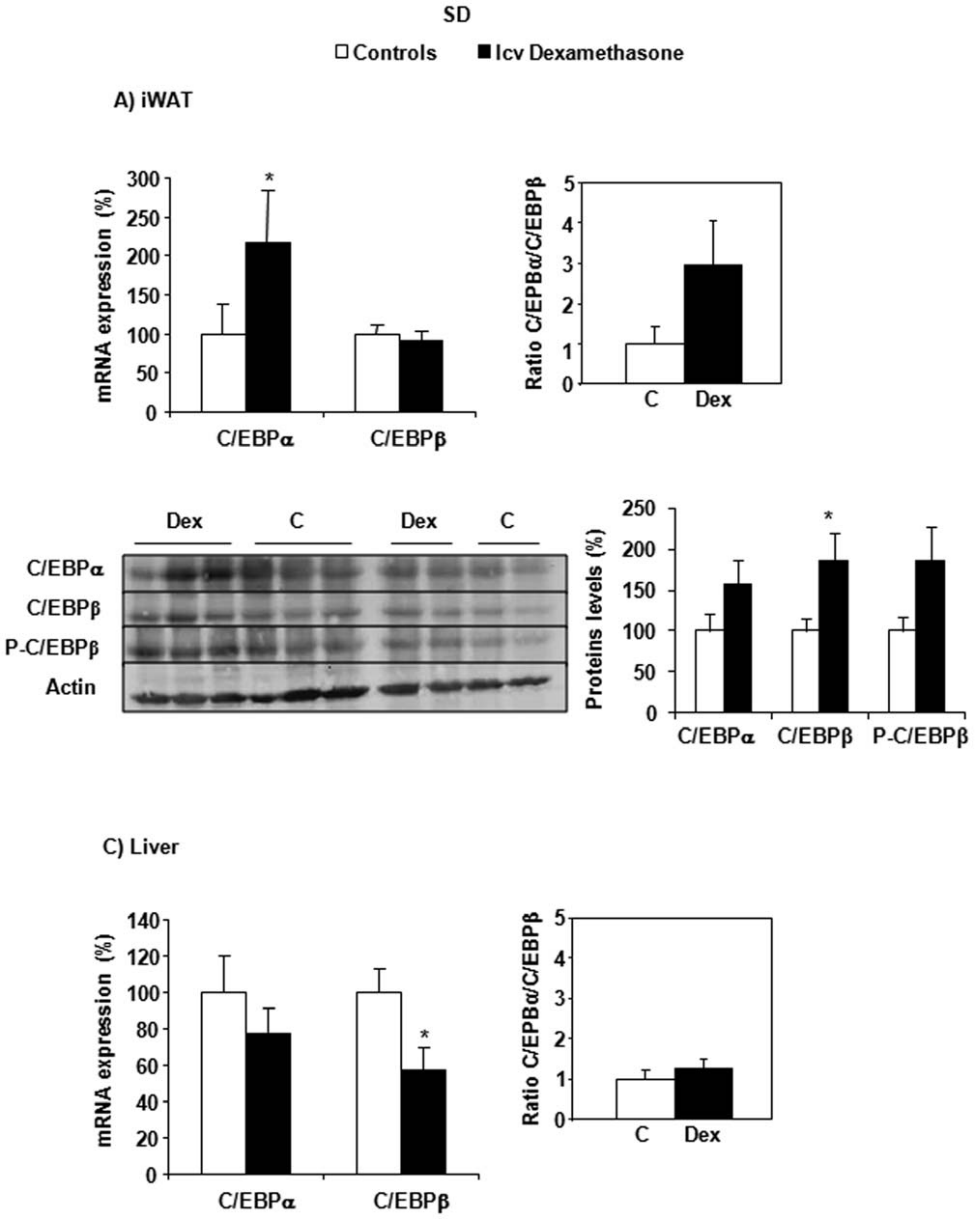
C/EBPα, C/EBPβ mRNA and protein expression in iWAT (A) and in the liver (C) of SD rats. Effects of central (icv) dexamethasone infusion for 2 days. A and C) Results are expressed as percent of relative mRNA expression compared to that obtained in control rats (100%). The analysis was performed in duplicate and the results were normalized with RPS29, *β*-actin and GAPDH expression. Mean ± SEM of *n* = 7–8 rats per group. B) Representative Western blots of C/EBPα, C/EBPβ and phosphorylated C/EBPβ-ser^105^ (n = 5 per group). Quantification was performed using the ChemiDoc™ XRS and the Quantity One™ software. *P<0.05 compared to vehicle-infused control rats (Student's *t* test).

Recent evidence suggests that the expression ratio of the transcription factors CCAAT enhancer binding protein (C/EBP)α to C/EBPβ might be important for the control of 11β-HSD1 expression [39], [40], [41], [42]. The expression of these two transcription factors was therefore characterized in iWAT and the liver of control and dexamethasone-treated SD rats. First, and as depicted in Fig. 6A, a two-fold increase in C/EBPα mRNA expression was observed in iWAT, in response to central dexamethasone infusion (*P* = 0.043), while C/EBPβ mRNA expression was unaltered by the treatment. This C/EBPα mRNA increase was highly correlated with the expression of resistin (r^2^ = 0.905, *P* = 0.001). When calculating the gene expression ratio of C/EBPα to C/EBPβ, an almost three-fold, although not significant increase was obtained in dexamethasone-treated animals (Fig. 6A). With regard to protein expression measured by Western blot analysis, a though 1.6 fold increase in C/EBPα was observed in the dexamethasone-treated group (Fig. 6B). In this group, both C/EBPβ and P-C/EBPβ expressions similarly increased by 1.86 folds. The resulting ratio between C/EBPα and C/EBPβ was unaltered by dexamethasone infusion at the protein level.

With regard to the liver, central dexamethasone administration induced a decreased C/EBPβ mRNA expression (*P* = 0.035), with no change in C/EBPα (Fig. 6C). The ratios of C/EBPα to C/EBPβ mRNA levels was comparable in the livers of both groups.

### Effect of peripheral dexamethasone infusion in SD rats

**Figure 7:**
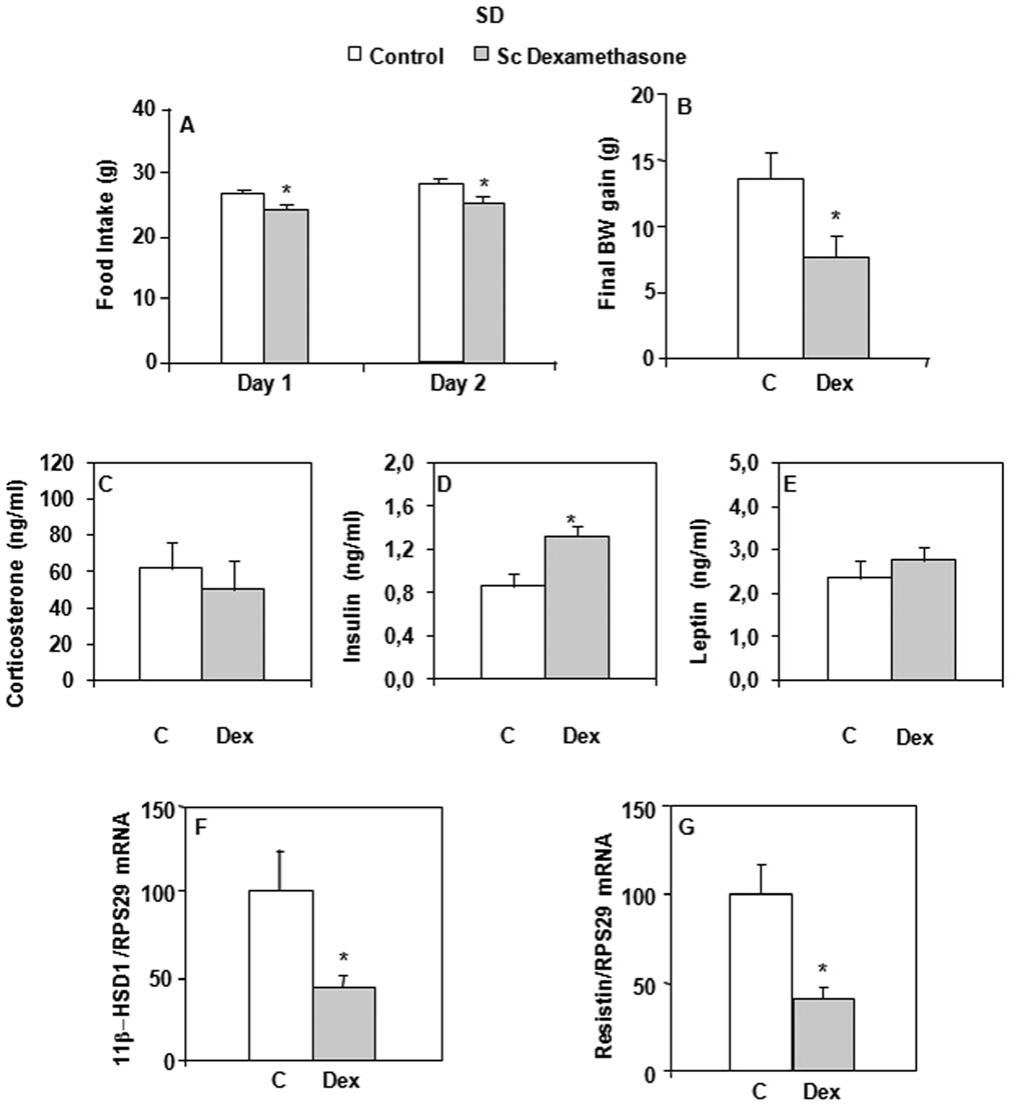
Food intake, body weight gain, corticosterone, insulin, leptin levels, 11β-HSD1 and resistin mRNA expression in SD rats. Effects of peripheral (sc) dexamethasone infusion for 3 days. A) Results are expressed as food ingested (in grams) each day of peripheral infusion. B) Results represent body weight gain (in grams) measured after 2 days of treatment. C to E) Plasma levels of corticosterone, insulin and leptin. F and G) Results are expressed as percent of relative mRNA expression compared to that obtained in control rats (100%). The analysis was performed in duplicate and the results were normalized with RPS29 expression. Mean ± SEM of *n* = 8–9 rats per group. *P<0.05 compared to vehicle-infused control rats (Student's *t* test).

Finally, in order to investigate the potential occurrence of dexamethasone leakage from the cerebrospinal fluid into the peripheral circulation, we infused 5 µg/day of dexamethasone subcutaneously (sc) during 2 days in SD rats. This treatment led to significant decreases in both daily food intake and body weight gain, compared with control rats receiving vehicle (Figs. 7A and 7B). Plasma corticosterone, leptin, glucose and TG levels were unaltered, while plasma insulin (*P* = 0.009) and NEFA (*P* = 0.024) concentrations were elevated (Figs. 7C–E and Table 1). The fact that, under these experimental conditions, dexamethasone infusion failed to decrease corticosterone levels suggested that the synthetic GC did not get into the brain. This was examined by determining the hypothalamic CRH mRNA expression, which was indeed unaltered by the treatment (100.0±14.9 and 90.0±20.4% for control and dexamethasone-treated rats, respectively, NS).

In contrast to what observed after icv infusion, 11β-HSD1 (*P* = 0.024) (Fig. 7F) and resistin (*P* = 0.028) (Fig. 7G) mRNA expression was lowered in iWAT of dexamethasone-treated rats. In the liver, neither the mRNA expression of 11β-HSD1, H6PDH, nor the mRNA or protein expression levels of PEPCK was modified. Finally, C/EBPα and C/EBPβ mRNA expression in iWAT or the liver was unaltered by this dose of peripheral dexamethasone administration (data not shown).

## Discussion

The present study aimed at determining the influence of short-term central glucocorticoid administration on local 11β-HSD expression in adipose tissue and in the liver to evaluate whether metabolic consequences of such a treatment, *i.e.* increased body weight gain and specific skeletal muscle insulin resistance [24], [25] may involve tissue-specific alterations.

As expected, centrally dexamethasone-infused rats displayed hypocorticosteronemia as a result of forcing the feedback inhibition of the HPA axis. The treatment also rapidly induced an increase in body weight gain due to increased food consumption during the diurnal period. Plasma leptin levels were significantly elevated, as was the mRNA expression of lipogenic enzymes such as FAS, suggesting the occurrence of increased lipid storage in subcutaneous and intraperitoneal white adipose tissue depots. Moreover, indirect calorimetry revealed a decreased rate of lipid oxidation in dexamethasone-treated rats during the diurnal period, together with indices of decreased energy expenditure and thermogenesis. In the Wistar strain exhibiting a high propensity to develop age-dependent obesity [36], the dexamethasone-induced increase in FAS expression was also accompanied by decreased expression of enzymes involved in lipolysis and lipid utilization. Altogether, these results confirm and extend our previous observations suggesting that short-term icv dexamethasone infusion in rats reproduces the initial metabolic defects occurring in obesity.

Analyzing the mRNA expression of 11β-HSD1, a differential regulation pattern was observed depending on the tissue and the strain. In obesity-prone Wistar rats [36], there was a marked dexamethasone-induced increase in 11β-HSD1 mRNA in inguinal white adipose tissue (iWAT). That was less obvious in SD animals known to be more heterogeneous in the face of diet-induced obesity [37], [38]. However in SD rats, a mild increase in 11β-HSD1 protein level was observed in the epididymal depot (eWAT). In addition to such a strain difference, it is also conceivable that regulation of 11β-HSD1 is transcriptional or translational, partly depending on its gene expression level. This is suggested by the observation that, under basal conditions, the expression of the enzyme was higher in intraperitoneal (eWAT) than in subcutaneous (iWAT) depots. It could thus be proposed that transcriptional 11β-HSD1 regulation is occurring when the enzyme is poorly expressed, while translational modulation occurs under high expression conditions.

Interestingly, the expression profiles of 11β-HSD1 and H6PDH, an enzyme determining the reductase activity of 11β-HSD1 [43], [44], [45], were similar and correlated within a given tissue and strain. As a whole, the results suggest enhanced 11β-HSD1 activity and activation of intracellular GCs in subcutaneous adipose depots of Wistar and SD rats. It should however be pointed out that these changes are only relevant if the plasma levels of 11-dehydrocorticosterone, the substrate for 11β-HSD1 enzymatic activity, are not decreased by dexamethasone infusion. To examine this issue, preliminary data were obtained by measuring both plasma corticosterone and 11-dehydrocorticosterone using LC-MSMS. They showed that the plasma concentrations of both compounds were unaffected by the dexamethasone treatment. Although these data need to be confirmed, they suggest that the availability of the substrate for 11β-HSD1 is not impaired by dexamethasone infusion.

Increased 11β-HSD1 activity and activation of adipose tissue GC production could be of importance, given the fact that these hormones have clear effects in adipose tissue, where they markedly potentiate the insulin effect on triglyceride uptake via the stimulation of lipoprotein lipase activity [46], while also promoting adipocyte differentiation [47]. Moreover, the observation that mice with adipose tissue 11β-HSD1 overexpression develop a visceral obesity syndrome with accompanying metabolic defects [19], [20] suggests that increased adipose tissue 11β-HSD1 expression might play a role in the development of obesity. Along this line, it should be noted that increased adipose tissue 11β-HSD1 expression was also observed in rodent models of genetic obesity [48], as well as in high fat fed mice [49].

Knowing that C/EBPs seem to play a key role in the activation of 11β-HSD1 and lipid storage-related genes in adipocytes [39], [42], [50], [51], the expression of C/EBPα and C/EBPβ, was determined. C/EBPα was indeed described as a potent activator of 11β-HSD1 and resistin [52], while C/EBPβ was described as a key mediator of 11β-HSD1 expression [41]. Accordingly, we observed that C/EBPα and resistin were overexpressed in inguinal adipose tissue of dexamethasone-treated SD rats with a strong correlation between both parameters. This is of importance as resistin may represent one of the mechanisms underlying the occurrence of muscle insulin resistance observed in response to central GC administration [24], [53], [54].

The situation was quite different in the liver, where we observed decreased mRNA expression of 11β-HSD1, C/EBPβ and PEPCK in dexamethasone-treated SD rats, with a trend toward a decrease in 11β-HSD1 and no change in PEPCK protein level. The liver is another tissue in which GCs may affect glucose metabolism. Of note, liver-specific overexpression of 11β-HSD1 resulted in insulin resistance and hypertension, but, in contrast to adipose tissue overexpression, it did not promote obesity [55]. In accordance, antisense-mediated inhibition of 11β-HSD1 in mice fed a Western-type diet resulted in improved plasma and hepatic lipid levels and reduced hepatic lipogenesis [56]. Decreased hepatic but enhanced adipose tissue 11β-HSD1 expression and activity were measured in genetically obese Zucker rats, *ob/ob* mice [5], [6], [20], [57], as well as in obese humans [16]. This might represent the establishment of a partial compensatory mechanism aimed at resisting hyperglycemia [10] by increasing local insulin sensitivity [58]. Interestingly, such an adaptation was only observed in the most obesity resistant strain, the SD rat after 2 days of dexamethasone treatment. Of note is the observation that a similar mechanism has been described in Wistar rats after 3 weeks of high fat feeding [8]. It is therefore possible that the dexamethasone treatment used in the present study was not long enough to induce this pathway in Wistar rats.

Regarding the mechanisms that could be implicated, one of the first questions posed is whether these changes are centrally elicited or whether they result from leakage of dexamethasone from the ventricular system into the peripheral circulation. In contrast to the icv treatment, peripheral dexamethasone treatment lowered food intake and body weight gain, but resulted in hyperinsulinemia and elevated plasma NEFA levels. The latter observation is in line with the lipolysis-inducing effects of GCs. These data favor the view that the icv dexamethasone-induced effects observed in our study are centrally elicited. This is also in keeping with data showing that central dexamethasone infusion decreases the hypothalamic content of CRH, while increasing that of NPY [25], a peptide known to promote the development of hyperphagia, vagus nerve-mediated basal insulin output and fat storage upon central infusion in normal rats [25], [59]. Finally, an involvement of insulin and corticosterone in the 11β-HSD1 changes observed could be considered, given their respective transcriptional inhibitory and stimulatory effects described *in vitro* [60], [61], [62], [63], [64]. However, in the present *in vivo* study, these hormones are unlikely to play a role, given that the changes in expression are tissue-specific and that the prevailing low levels of circulating corticosterone should allow unopposed inhibitory insulin effects on 11β-HSD1, which is not observed in adipose tissue.

Taken together, these results show that acute central glucocorticoid administration causes differential effects on local GC activation. We propose that in this acute model, the GC-dependent metabolic disturbances first appear in adipose tissue overexpressing resistin and in skeletal muscles exhibiting marked insulin resistance [24]. The concomitant decreased GC activation in the liver would initially limit the metabolic disturbances accompanying the development of increased body weight. The particular importance of GC activation in adipose tissue for the development of metabolic dysfunctions is further strengthened by the observations that administration of specific 11β-HSD1 inhibitors resulted in significant improvement of glucose and lipid profiles, together with a weight reduction, provided that 11β-HSD1 activity was decreased in adipose tissue, rather than in the liver [65]. The precise mechanisms by which GCs affect peripheral metabolism by exerting a central action need to be further elucidated.

## Supporting Information

Table S1 Primer sequences used for real-time PCR.
(DOCX)
