# Supplementary material for: Central Glucocorticoid Administration Promotes Weight Gain and Increased 11β-Hydroxysteroid Dehydrogenase Type 1 Expression in White Adipose Tissue
Source: PLoS One. 2012 Mar 30;7(3):e34002. doi: 10.1371/journal.pone.0034002 (PMC3316512; doi:10.1371/journal.pone.0034002)
Supplement: Table S1 — Primer sequences used for real-time PCR. (DOCX) [file pone.0034002.s001.docx]

**Table S1.** Primer sequences used for real-time PCR

| Gene | Accession Number | Primers |
| --- | --- | --- |
| 11-ßHSD1  11-ßHSD2  36b4  ACCα  ATGL  β-actin  C/EBPα  C/EBPß  CPT1  CRH  FAS  GAPDH  GR  H6PDH  HSL  LPL  PEPCK  Resistin  RPS29 | NM017080  NM017081  AC130745  NM022193  NM025802  NM031144  NM012524  NM024125  NM031559  NM031019  NM017332  NM017008  M14053  NM001106698  NM012859  NM012598  NM198780  NM144741  NM012876 | 5’-TCTGTGAGCCCTACCCACAAA / 5’-CATGGTGAGGTGTACCCCAAA  5’-CTGGCTGGTGCTGGCTGGA / 5’-CCGTGAAGCCCATGCATCC  5’-TTCCCACTGGCTGAAAAGGT / 5’-CGCAGCCGCAAATGC  5’-ACCTCAACCACTACGGCATGA / 5’-AGGTGGTGTGAAGGCGTTGT  5’-GGCCATGATGGTGCCCTATA / 5’-CCAACAAGCGGATGGTGAA  Provided by Applied Biosystems  5’-GAGCCGAGATAAAGCCAACAG / 5’-TCATTGTCACTGGTCAACTCCAA  Provided by Applied Biosystems  5’-CGGTTCAAGAATGGCATCATC / 5’-TCACACCCACCACCACGATA  5’-GGATCTCACCTTCCACCTTCT / 5’-TTCCTGTTGCTGTGAGCTTG  5’-GGACATGGTCACAGACGATGAC / 5’-CGTCGAACTTGGACAGATCCTT  5’-cagtgccagcctcgtctca / 5’-tggtaaccaggcgtccgata  5’-ggaggagctacagtcaaggtttct / 5’-ctgcttggaatctgcctgaga  5’-GATGAGCGTTGTGTCCCTCT / 5’-GGCATGGGGTGGATATTG  5’-CCCCGAGATGTCACAGTCAAT / 5’-GAATTCCCGGATCGCAGAA  5’-GGCATACAGGTGCAATTCCA / 5’-CGTCGAACTTGGACAGATCCTT  5’-GGCATCCCTGTGACTTTTTCA / 5’-CTTTCGATCCTGGCCACATC  5’-CTACTGCCAGCTGCAATGAA / 5’-AGGGCAAGCTCAGTTCTCAA  5’-GCCAGGGTTCTCGCTCTTG / 5’-GGCACATGTTCAGCCCGTAT |
